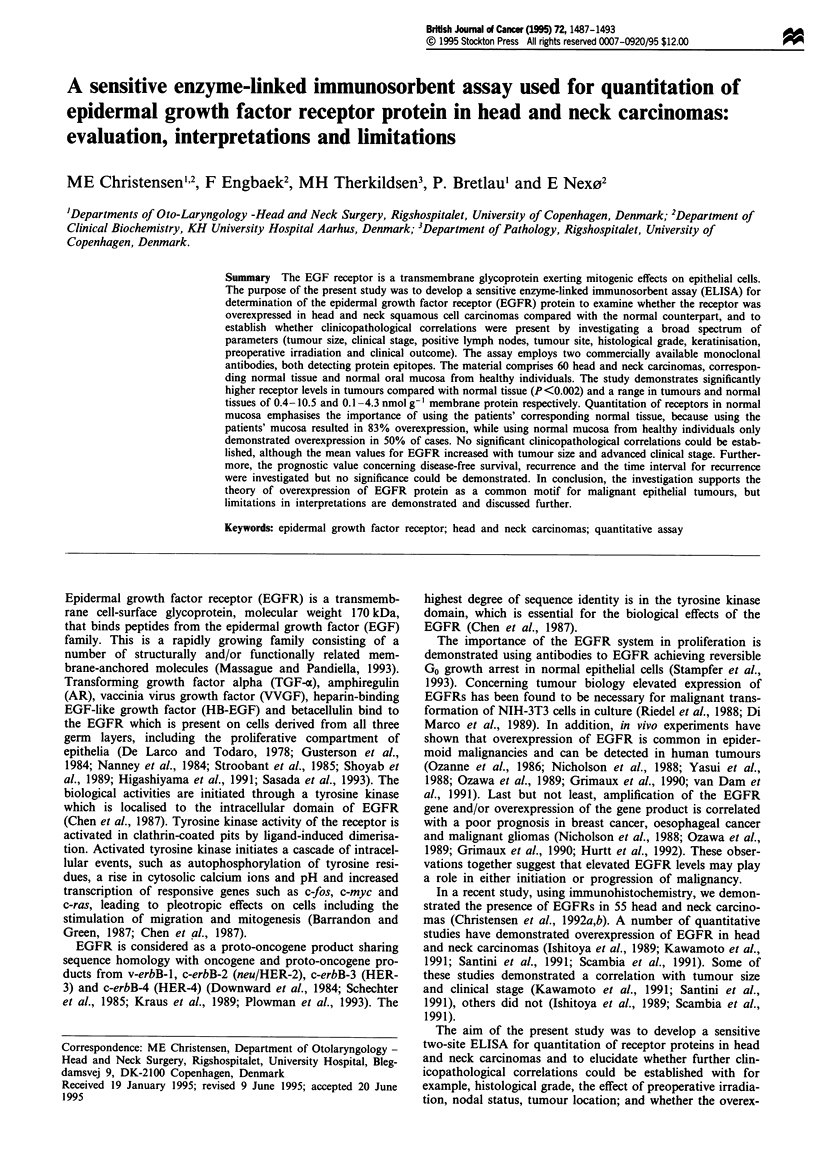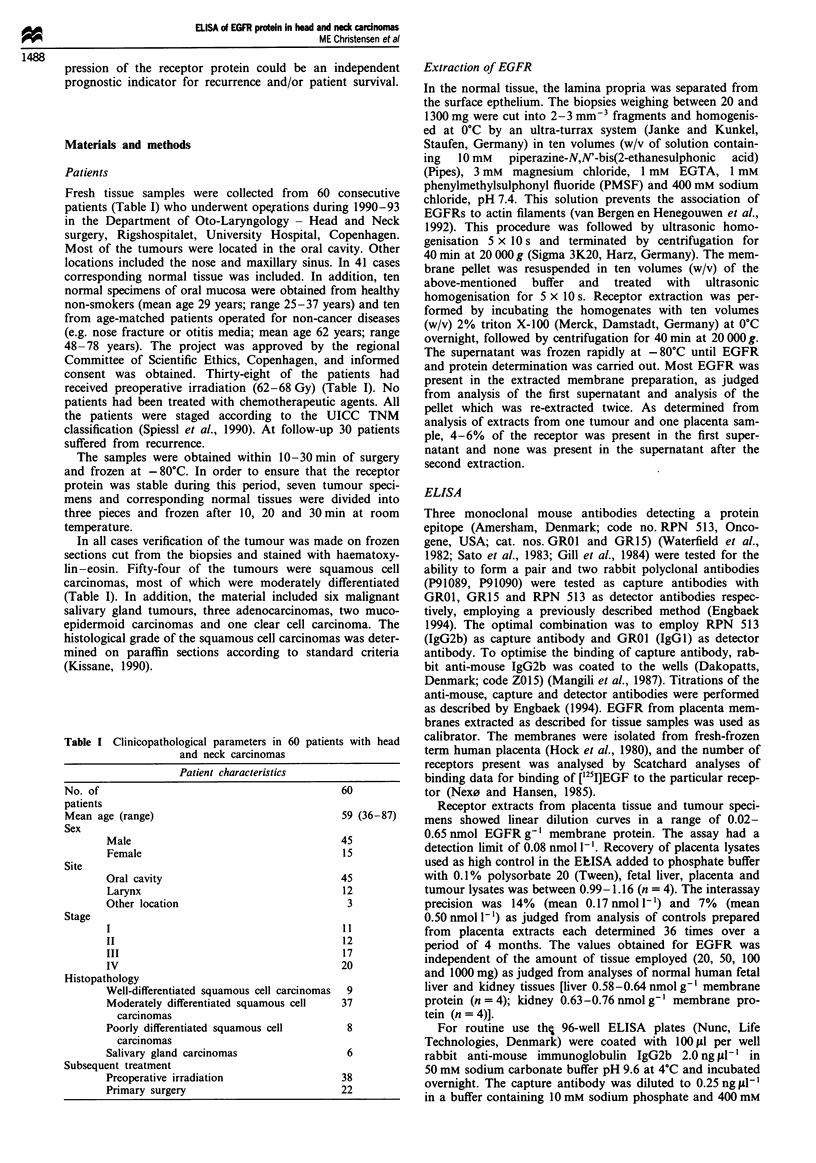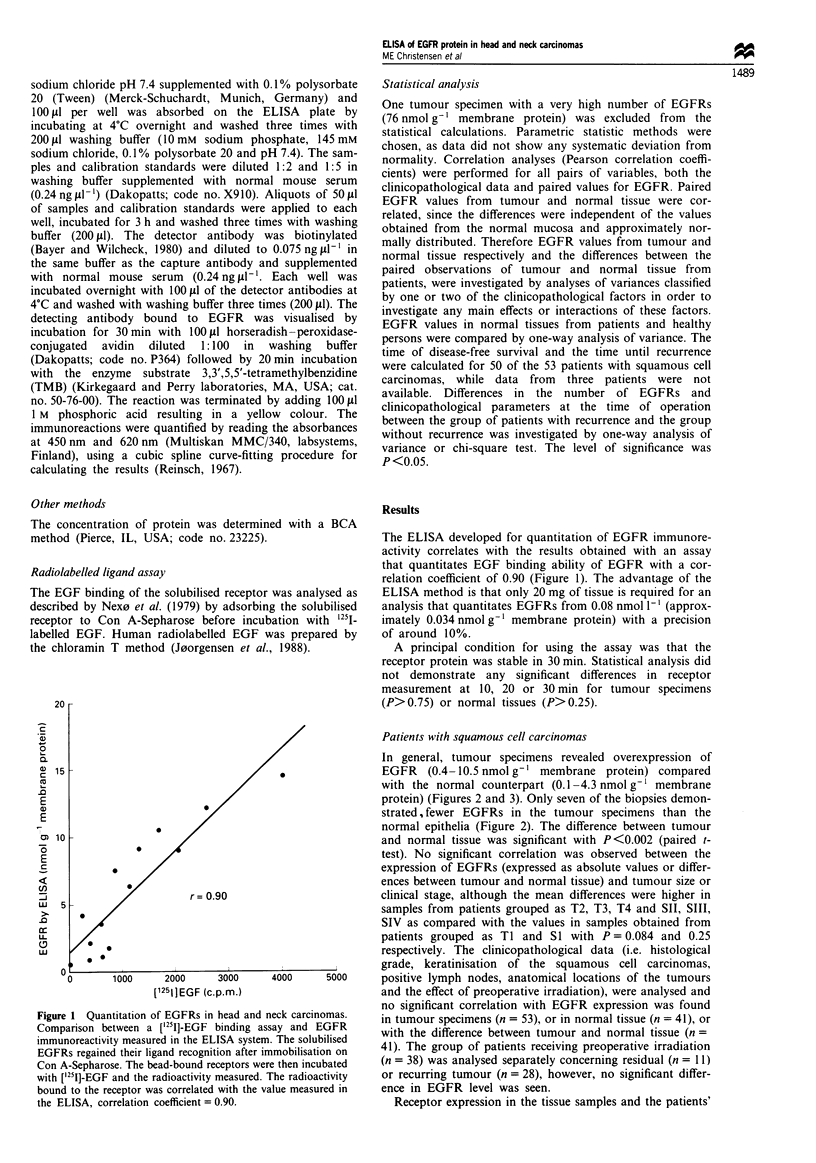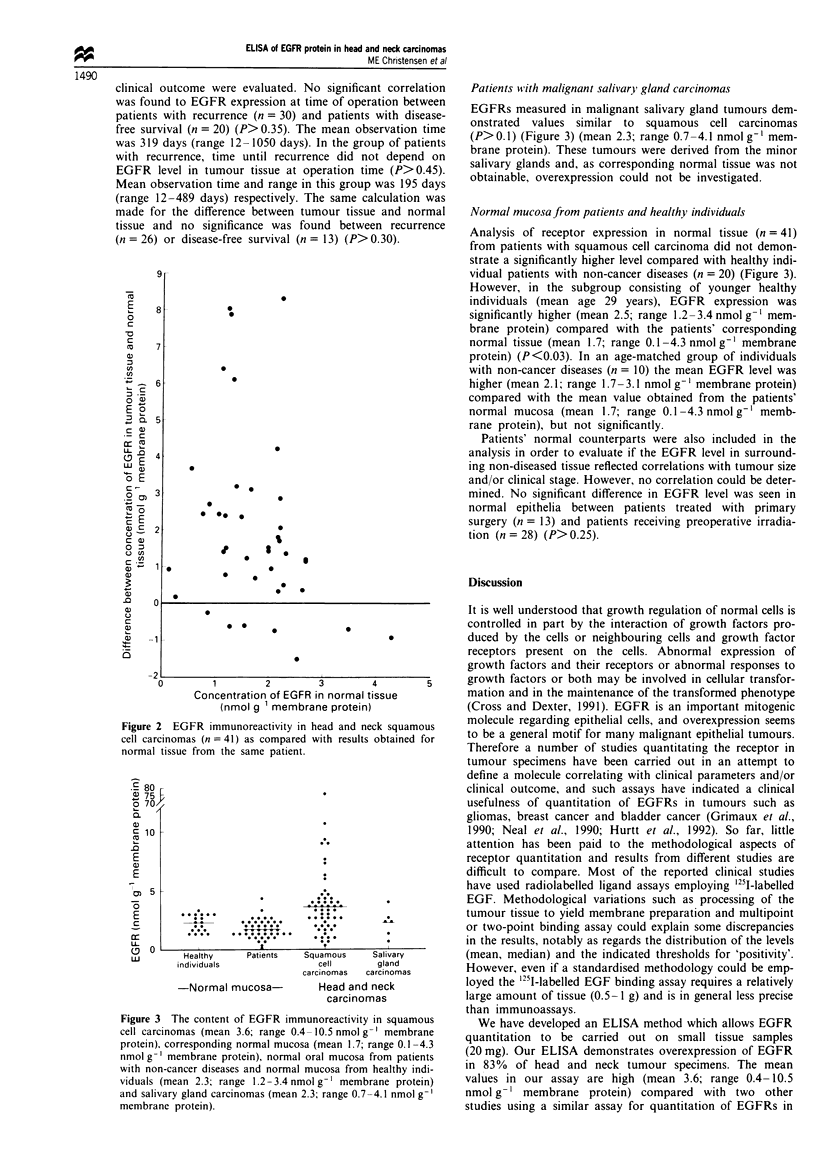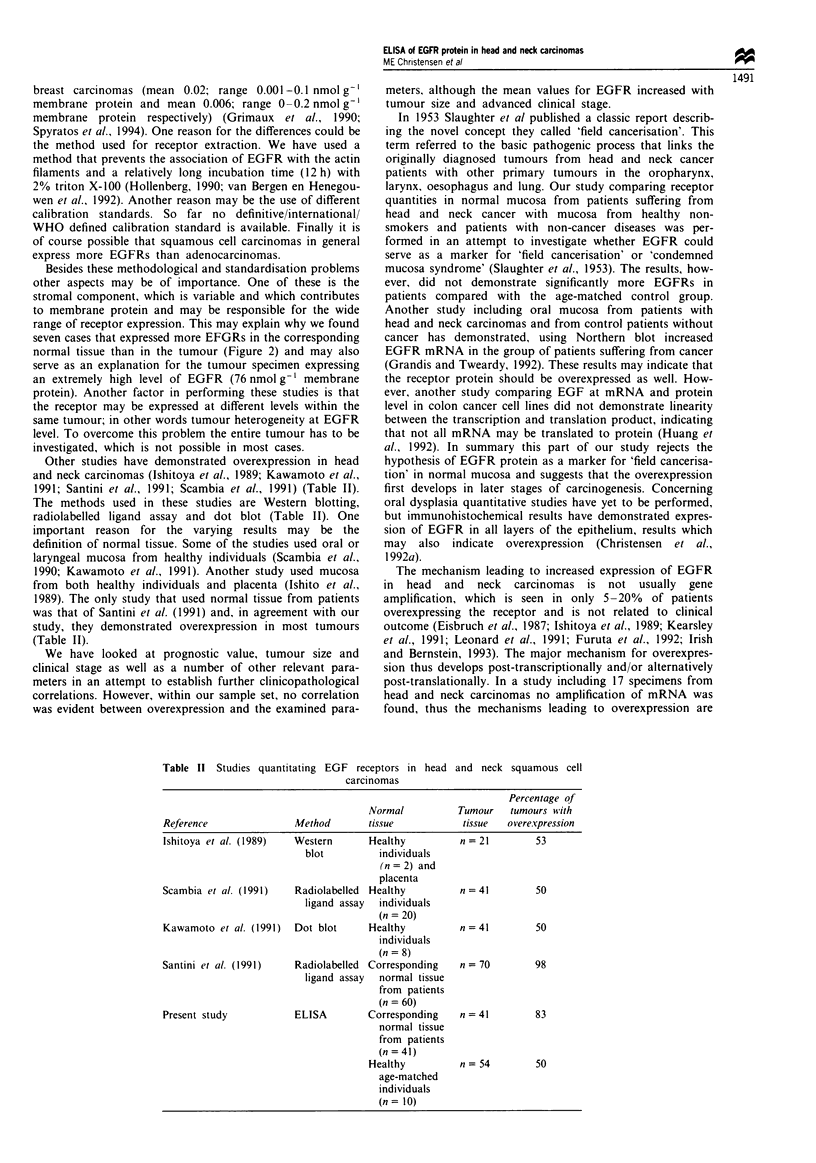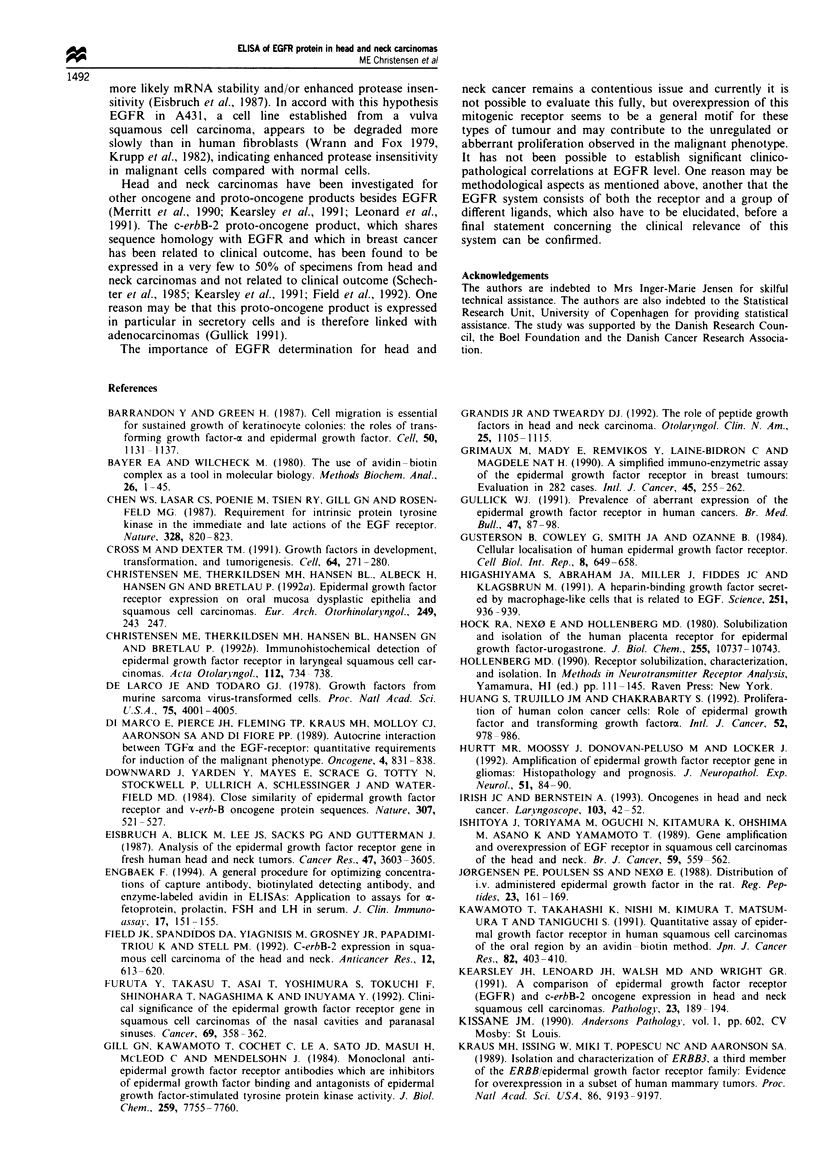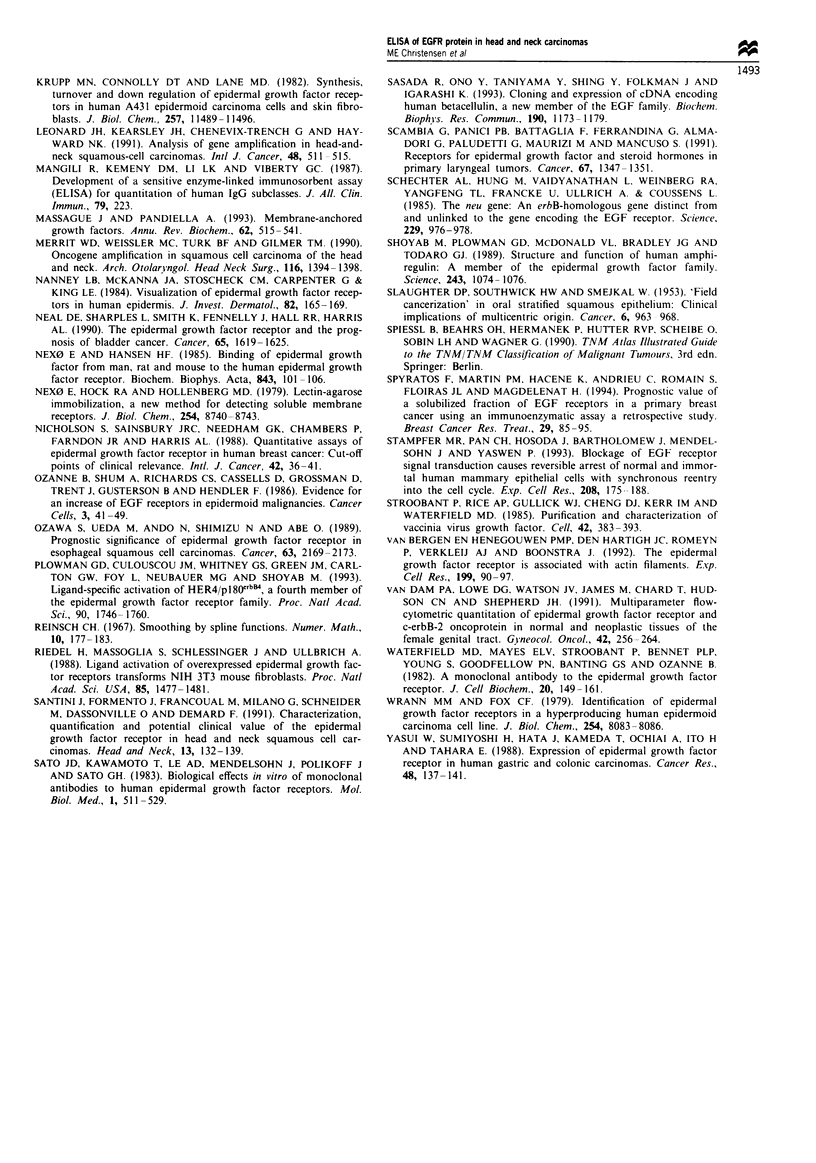# A sensitive enzyme-linked immunosorbent assay used for quantitation of epidermal growth factor receptor protein in head and neck carcinomas: evaluation, interpretations and limitations.

**DOI:** 10.1038/bjc.1995.534

**Published:** 1995-12

**Authors:** M. E. Christensen, F. Engbaek, M. H. Therkildsen, P. Bretlau, E. Nexø

**Affiliations:** Department of Oto-Laryngology-Head and Neck Surgery, Rigshospitalet, University of Copenhagen, Denmark.

## Abstract

The EGF receptor is a transmembrane glycoprotein exerting mitogenic effects on epithelial cells. The purpose of the present study was to develop a sensitive enzyme-linked immunosorbent assay (ELISA) for determination of the epidermal growth factor receptor (EGFR) protein to examine whether the receptor was overexpressed in head and neck squamous cell carcinomas compared with the normal counterpart, and to establish whether clinicopathological correlations were present by investigating a broad spectrum of parameters (tumour size, clinical stage, positive lymph nodes, tumour site, histological grade, keratinisation, preoperative irradiation and clinical outcome). The assay employs two commercially available monoclonal antibodies, both detecting protein epitopes. The material comprises 60 head and neck carcinomas, corresponding normal tissue and normal oral mucosa from healthy individuals. The study demonstrates significantly higher receptor levels in tumours compared with normal tissue (P < 0.002) and a range in tumours and normal tissues of 0.4-10.5 and 0.1-4.3 nmol g-1 membrane protein respectively. Quantitation of receptors in normal mucosa emphasises the importance of using the patients' corresponding normal tissue, because using the patients' mucosa resulted in 83% overexpression, while using normal mucosa from healthy individuals only demonstrated overexpression in 50% of cases. No significant clinicopathological correlations could be established, although the mean values for EGFR increased with tumour size and advanced clinical stage. Furthermore, the prognostic value concerning disease-free survival, recurrence and the time interval for recurrence were investigated but no significance could be demonstrated. In conclusion, the investigation supports the theory of overexpression of EGFR protein as a common motif for malignant epithelial tumours, but limitations in interpretations are demonstrated and discussed further.


British Journal of Cancer (1995) 72, 1487-1493
© 1995 Stockton Press All rights reserved 0007-0920/95 $12.00

### A sensitive enzyme-linked immunosorbent assay used for quantitation of epidermal growth factor receptor protein in head and neck carcinomas: evaluation, interpretations and limitations

ME Christensen[1,2], F Engbaek[2], MH Therkildsen[3], P. Bretlau[1] and E Nexø[2]

[1]Departments of Oto-Laryngology -Head and Neck Surgery, Rigshospitalet, University of Copenhagen, Denmark; [2]Department of Clinical Biochemistry, KH University Hospital Aarhus, Denmark; [3]Department of Pathology, Rigshospitalet, University of Copenhagen, Denmark.

**Summary** The EGF receptor is a transmembrane glycoprotein exerting mitogenic effects on epithelial cells. The purpose of the present study was to develop a sensitive enzyme-linked immunosorbent assay (ELISA) for determination of the epidermal growth factor receptor (EGFR) protein to examine whether the receptor was overexpressed in head and neck squamous cell carcinomas compared with the normal counterpart, and to establish whether clinicopathological correlations were present by investigating a broad spectrum of parameters (tumour size, clinical stage, positive lymph nodes, tumour site, histological grade, keratinisation, preoperative irradiation and clinical outcome). The assay employs two commercially available monoclonal antibodies, both detecting protein epitopes. The material comprises 60 head and neck carcinomas, corresponding normal tissue and normal oral mucosa from healthy individuals. The study demonstrates significantly higher receptor levels in tumours compared with normal tissue ($P<0.002$) and a range in tumours and normal tissues of 0.4–10.5 and 0.1–4.3 nmol $g^{-1}$ membrane protein respectively. Quantitation of receptors in normal mucosa emphasises the importance of using the patients' corresponding normal tissue, because using the patients' mucosa resulted in 83% overexpression, while using normal mucosa from healthy individuals only demonstrated overexpression in 50% of cases. No significant clinicopathological correlations could be established, although the mean values for EGFR increased with tumour size and advanced clinical stage. Furthermore, the prognostic value concerning disease-free survival, recurrence and the time interval for recurrence were investigated but no significance could be demonstrated. In conclusion, the investigation supports the theory of overexpression of EGFR protein as a common motif for malignant epithelial tumours, but limitations in interpretations are demonstrated and discussed further.

**Keywords:** epidermal growth factor receptor; head and neck carcinomas; quantitative assay

Epidermal growth factor receptor (EGFR) is a transmembrane cell-surface glycoprotein, molecular weight 170 kDa, that binds peptides from the epidermal growth factor (EGF) family. This is a rapidly growing family consisting of a number of structurally and/or functionally related membrane-anchored molecules (Massague and Pandiella, 1993). Transforming growth factor alpha (TGF-α), amphiregulin (AR), vaccinia virus growth factor (VVGF), heparin-binding EGF-like growth factor (HB-EGF) and betacellulin bind to the EGFR which is present on cells derived from all three germ layers, including the proliferative compartment of epithelia (De Larco and Todaro, 1978; Gusterson *et al.*, 1984; Nanney *et al.*, 1984; Stroobant *et al.*, 1985; Shoyab *et al.*, 1989; Higashiyama *et al.*, 1991; Sasada *et al.*, 1993). The biological activities are initiated through a tyrosine kinase which is localised to the intracellular domain of EGFR (Chen *et al.*, 1987). Tyrosine kinase activity of the receptor is activated in clathrin-coated pits by ligand-induced dimerisation. Activated tyrosine kinase initiates a cascade of intracellular events, such as autophosphorylation of tyrosine residues, a rise in cytosolic calcium ions and pH and increased transcription of responsive genes such as c-*fos*, c-*myc* and c-*ras*, leading to pleotropic effects on cells including the stimulation of migration and mitogenesis (Barrandon and Green, 1987; Chen *et al.*, 1987).

EGFR is considered as a proto-oncogene product sharing sequence homology with oncogene and proto-oncogene products from v-*erb*B-1, c-*erb*B-2 (*neu*/HER-2), c-*erb*B-3 (HER-3) and c-*erb*B-4 (HER-4) (Downward *et al.*, 1984; Schechter *et al.*, 1985; Kraus *et al.*, 1989; Plowman *et al.*, 1993). The highest degree of sequence identity is in the tyrosine kinase domain, which is essential for the biological effects of the EGFR (Chen *et al.*, 1987).

The importance of the EGFR system in proliferation is demonstrated using antibodies to EGFR achieving reversible $G_0$ growth arrest in normal epithelial cells (Stampfer *et al.*, 1993). Concerning tumour biology elevated expression of EGFRs has been found to be necessary for malignant transformation of NIH-3T3 cells in culture (Riedel *et al.*, 1988; Di Marco *et al.*, 1989). In addition, *in vivo* experiments have shown that overexpression of EGFR is common in epidermoid malignancies and can be detected in human tumours (Ozanne *et al.*, 1986; Nicholson *et al.*, 1988; Yasui *et al.*, 1988; Ozawa *et al.*, 1989; Grimaux *et al.*, 1990; van Dam *et al.*, 1991). Last but not least, amplification of the EGFR gene and/or overexpression of the gene product is correlated with a poor prognosis in breast cancer, oesophageal cancer and malignant gliomas (Nicholson *et al.*, 1988; Ozawa *et al.*, 1989; Grimaux *et al.*, 1990; Hurtt *et al.*, 1992). These observations together suggest that elevated EGFR levels may play a role in either initiation or progression of malignancy.

In a recent study, using immunohistochemistry, we demonstrated the presence of EGFRs in 55 head and neck carcinomas (Christensen *et al.*, 1992*a*,*b*). A number of quantitative studies have demonstrated overexpression of EGFR in head and neck carcinomas (Ishitoya *et al.*, 1989; Kawamoto *et al.*, 1991; Santini *et al.*, 1991; Scambia *et al.*, 1991). Some of these studies demonstrated a correlation with tumour size and clinical stage (Kawamoto *et al.*, 1991; Santini *et al.*, 1991), others did not (Ishitoya *et al.*, 1989; Scambia *et al.*, 1991).

The aim of the present study was to develop a sensitive two-site ELISA for quantitation of receptor proteins in head and neck carcinomas and to elucidate whether further clinicopathological correlations could be established with for example, histological grade, the effect of preoperative irradiation, nodal status, tumour location; and whether the overex-

Correspondence: ME Christensen, Department of Otolaryngology – Head and Neck Surgery, Rigshospitalet, University Hospital, Blegdamsvej 9, DK-2100 Copenhagen, Denmark
Received 19 January 1995; revised 9 June 1995; accepted 20 June 1995

pression of the receptor protein could be an independent prognostic indicator for recurrence and/or patient survival.

### Materials and methods

### Patients

Fresh tissue samples were collected from 60 consecutive patients (Table I) who underwent operations during 1990–93 in the Department of Oto-Laryngology – Head and Neck surgery, Rigshospitalet, University Hospital, Copenhagen. Most of the tumours were located in the oral cavity. Other locations included the nose and maxillary sinus. In 41 cases corresponding normal tissue was included. In addition, ten normal specimens of oral mucosa were obtained from healthy non-smokers (mean age 29 years; range 25–37 years) and ten from age-matched patients operated for non-cancer diseases (e.g. nose fracture or otitis media; mean age 62 years; range 48–78 years). The project was approved by the regional Committee of Scientific Ethics, Copenhagen, and informed consent was obtained. Thirty-eight of the patients had received preoperative irradiation (62–68 Gy) (Table I). No patients had been treated with chemotherapeutic agents. All the patients were staged according to the UICC TNM classification (Spiessl *et al.*, 1990). At follow-up 30 patients suffered from recurrence.

The samples were obtained within 10–30 min of surgery and frozen at −80°C. In order to ensure that the receptor protein was stable during this period, seven tumour specimens and corresponding normal tissues were divided into three pieces and frozen after 10, 20 and 30 min at room temperature.

In all cases verification of the tumour was made on frozen sections cut from the biopsies and stained with haematoxylin–eosin. Fifty-four of the tumours were squamous cell carcinomas, most of which were moderately differentiated (Table I). In addition, the material included six malignant salivary gland tumours, three adenocarcinomas, two mucoepidermoid carcinomas and one clear cell carcinoma. The histological grade of the squamous cell carcinomas was determined on paraffin sections according to standard criteria (Kissane, 1990).

**Table I** Clinicopathological parameters in 60 patients with head and neck carcinomas

| Patient characteristics | | |
|---|---|---|
| No. of patients | | 60 |
| Mean age (range) | | 59 (36–87) |
| Sex | | |
| | Male | 45 |
| | Female | 15 |
| Site | | |
| | Oral cavity | 45 |
| | Larynx | 12 |
| | Other location | 3 |
| Stage | | |
| | I | 11 |
| | II | 12 |
| | III | 17 |
| | IV | 20 |
| Histopathology | | |
| | Well-differentiated squamous cell carcinomas | 9 |
| | Moderately differentiated squamous cell carcinomas | 37 |
| | Poorly differentiated squamous cell carcinomas | 8 |
| | Salivary gland carcinomas | 6 |
| Subsequent treatment | | |
| | Preoperative irradiation | 38 |
| | Primary surgery | 22 |

### Extraction of EGFR

In the normal tissue, the lamina propria was separated from the surface epthelium. The biopsies weighing between 20 and 1300 mg were cut into 2–3 $mm^{-3}$ fragments and homogenised at 0°C by an ultra-turrax system (Janke and Kunkel, Staufen, Germany) in ten volumes (w/v of solution containing 10 mM piperazine-*N*,*N*′-bis(2-ethanesulphonic acid) (Pipes), 3 mM magnesium chloride, 1 mM EGTA, 1 mM phenylmethylsulphonyl fluoride (PMSF) and 400 mM sodium chloride, pH 7.4. This solution prevents the association of EGFRs to actin filaments (van Bergen en Henegouwen *et al.*, 1992). This procedure was followed by ultrasonic homogenisation 5 × 10 s and terminated by centrifugation for 40 min at 20 000 *g* (Sigma 3K20, Harz, Germany). The membrane pellet was resuspended in ten volumes (w/v) of the above-mentioned buffer and treated with ultrasonic homogenisation for 5 × 10 s. Receptor extraction was performed by incubating the homogenates with ten volumes (w/v) 2% triton X-100 (Merck, Damstadt, Germany) at 0°C overnight, followed by centrifugation for 40 min at 20 000 *g*. The supernatant was frozen rapidly at −80°C until EGFR and protein determination was carried out. Most EGFR was present in the extracted membrane preparation, as judged from analysis of the first supernatant and analysis of the pellet which was re-extracted twice. As determined from analysis of extracts from one tumour and one placenta sample, 4–6% of the receptor was present in the first supernatant and none was present in the supernatant after the second extraction.

### ELISA

Three monoclonal mouse antibodies detecting a protein epitope (Amersham, Denmark; code no. RPN 513, Oncogene, USA; cat. nos. GR01 and GR15) (Waterfield *et al.*, 1982; Sato *et al.*, 1983; Gill *et al.*, 1984) were tested for the ability to form a pair and two rabbit polyclonal antibodies (P91089, P91090) were tested as capture antibodies with GR01, GR15 and RPN 513 as detector antibodies respectively, employing a previously described method (Engbaek 1994). The optimal combination was to employ RPN 513 (IgG2b) as capture antibody and GR01 (IgG1) as detector antibody. To optimise the binding of capture antibody, rabbit anti-mouse IgG2b was coated to the wells (Dakopatts, Denmark; code Z015) (Mangili *et al.*, 1987). Titrations of the anti-mouse, capture and detector antibodies were performed as described by Engbaek (1994). EGFR from placenta membranes extracted as described for tissue samples was used as calibrator. The membranes were isolated from fresh-frozen term human placenta (Hock *et al.*, 1980), and the number of receptors present was analysed by Scatchard analyses of binding data for binding of [$^{125}$I]EGF to the particular receptor (Nexø and Hansen, 1985).

Receptor extracts from placenta tissue and tumour specimens showed linear dilution curves in a range of 0.02–0.65 nmol EGFR $g^{-1}$ membrane protein. The assay had a detection limit of 0.08 nmol $l^{-1}$. Recovery of placenta lysates used as high control in the ELISA added to phosphate buffer with 0.1% polysorbate 20 (Tween), fetal liver, placenta and tumour lysates was between 0.99–1.16 ($n = 4$). The interassay precision was 14% (mean 0.17 nmol $l^{-1}$) and 7% (mean 0.50 nmol $l^{-1}$) as judged from analysis of controls prepared from placenta extracts each determined 36 times over a period of 4 months. The values obtained for EGFR was independent of the amount of tissue employed (20, 50, 100 and 1000 mg) as judged from analyses of normal human fetal liver and kidney tissues [liver 0.58–0.64 nmol $g^{-1}$ membrane protein ($n = 4$); kidney 0.63–0.76 nmol $g^{-1}$ membrane protein ($n = 4$)].

For routine use the 96-well ELISA plates (Nunc, Life Technologies, Denmark) were coated with 100 µl per well rabbit anti-mouse immunoglobulin IgG2b 2.0 ng $\mu l^{-1}$ in 50 mM sodium carbonate buffer pH 9.6 at 4°C and incubated overnight. The capture antibody was diluted to 0.25 ng $\mu l^{-1}$ in a buffer containing 10 mM sodium phosphate and 400 mM

sodium chloride pH 7.4 supplemented with 0.1% polysorbate 20 (Tween) (Merck-Schuchardt, Munich, Germany) and 100 µl per well was absorbed on the ELISA plate by incubating at 4°C overnight and washed three times with 200 µl washing buffer (10 mM sodium phosphate, 145 mM sodium chloride, 0.1% polysorbate 20 and pH 7.4). The samples and calibration standards were diluted 1:2 and 1:5 in washing buffer supplemented with normal mouse serum (0.24 ng $\mu l^{-1}$) (Dakopatts; code no. X910). Aliquots of 50 µl of samples and calibration standards were applied to each well, incubated for 3 h and washed three times with washing buffer (200 µl). The detector antibody was biotinylated (Bayer and Wilcheck, 1980) and diluted to 0.075 ng $\mu l^{-1}$ in the same buffer as the capture antibody and supplemented with normal mouse serum (0.24 ng $\mu l^{-1}$. Each well was incubated overnight with 100 µl of the detector antibodies at 4°C and washed with washing buffer three times (200 µl). The detecting antibody bound to EGFR was visualised by incubation for 30 min with 100 µl horseradish–peroxidase-conjugated avidin diluted 1:100 in washing buffer (Dakopatts; code no. P364) followed by 20 min incubation with the enzyme substrate 3,3′,5,5′-tetramethylbenzidine (TMB) (Kirkegaard and Perry laboratories, MA, USA; cat. no. 50-76-00). The reaction was terminated by adding 100 µl 1 M phosphoric acid resulting in a yellow colour. The immunoreactions were quantified by reading the absorbances at 450 nm and 620 nm (Multiskan MMC/340, labsystems, Finland), using a cubic spline curve-fitting procedure for calculating the results (Reinsch, 1967).

*Other methods*

The concentration of protein was determined with a BCA method (Pierce, IL, USA; code no. 23225).

*Radiolabelled ligand assay*

The EGF binding of the solubilised receptor was analysed as described by Nexø *et al.* (1979) by adsorbing the solubilised receptor to Con A-Sepharose before incubation with $^{125}$I-labelled EGF. Human radiolabelled EGF was prepared by the chloramin T method (Jøorgensen *et al.*, 1988).

**Figure 1** Quantitation of EGFRs in head and neck carcinomas. Comparison between a [$^{125}$I]-EGF binding assay and EGFR immunoreactivity measured in the ELISA system. The solubilised EGFRs regained their ligand recognition after immobilisation on Con A-Sepharose. The bead-bound receptors were then incubated with [$^{125}$I]-EGF and the radioactivity measured. The radioactivity bound to the receptor was correlated with the value measured in the ELISA, correlation coefficient = 0.90.

*Statistical analysis*

One tumour specimen with a very high number of EGFRs (76 nmol $g^{-1}$ membrane protein) was excluded from the statistical calculations. Parametric statistic methods were chosen, as data did not show any systematic deviation from normality. Correlation analyses (Pearson correlation coefficients) were performed for all pairs of variables, both the clinicopathological data and paired values for EGFR. Paired EGFR values from tumour and normal tissue were correlated, since the differences were independent of the values obtained from the normal mucosa and approximately normally distributed. Therefore EGFR values from tumour and normal tissue respectively and the differences between the paired observations of tumour and normal tissue from patients, were investigated by analyses of variances classified by one or two of the clinicopathological factors in order to investigate any main effects or interactions of these factors. EGFR values in normal tissues from patients and healthy persons were compared by one-way analysis of variance. The time of disease-free survival and the time until recurrence were calculated for 50 of the 53 patients with squamous cell carcinomas, while data from three patients were not available. Differences in the number of EGFRs and clinicopathological parameters at the time of operation between the group of patients with recurrence and the group without recurrence was investigated by one-way analysis of variance or chi-square test. The level of significance was $P<0.05$.

### Results

The ELISA developed for quantitation of EGFR immunoreactivity correlates with the results obtained with an assay that quantitates EGF binding ability of EGFR with a correlation coefficient of 0.90 (Figure 1). The advantage of the ELISA method is that only 20 mg of tissue is required for an analysis that quantitates EGFRs from 0.08 nmol $l^{-1}$ (approximately 0.034 nmol $g^{-1}$ membrane protein) with a precision of around 10%.

A principal condition for using the assay was that the receptor protein was stable in 30 min. Statistical analysis did not demonstrate any significant differences in receptor measurement at 10, 20 or 30 min for tumour specimens ($P>0.75$) or normal tissues ($P>0.25$).

*Patients with squamous cell carcinomas*

In general, tumour specimens revealed overexpression of EGFR (0.4–10.5 nmol $g^{-1}$ membrane protein) compared with the normal counterpart (0.1–4.3 nmol $g^{-1}$ membrane protein) (Figures 2 and 3). Only seven of the biopsies demonstrated, fewer EGFRs in the tumour specimens than the normal epithelia (Figure 2). The difference between tumour and normal tissue was significant with $P<0.002$ (paired *t*-test). No significant correlation was observed between the expression of EGFRs (expressed as absolute values or differences between tumour and normal tissue) and tumour size or clinical stage, although the mean differences were higher in samples from patients grouped as T2, T3, T4 and SII, SIII, SIV as compared with the values in samples obtained from patients grouped as T1 and S1 with $P = 0.084$ and 0.25 respectively. The clinicopathological data (i.e. histological grade, keratinisation of the squamous cell carcinomas, positive lymph nodes, anatomical locations of the tumours and the effect of preoperative irradiation), were analysed and no significant correlation with EGFR expression was found in tumour specimens ($n = 53$), or in normal tissue ($n = 41$), or with the difference between tumour and normal tissue ($n = 41$). The group of patients receiving preoperative irradiation ($n = 38$) was analysed separately concerning residual ($n = 11$) or recurring tumour ($n = 28$), however, no significant difference in EGFR level was seen.

Receptor expression in the tissue samples and the patients'

clinical outcome were evaluated. No significant correlation was found to EGFR expression at time of operation between patients with recurrence ($n = 30$) and patients with disease-free survival ($n = 20$) ($P > 0.35$). The mean observation time was 319 days (range 12–1050 days). In the group of patients with recurrence, time until recurrence did not depend on EGFR level in tumour tissue at operation time ($P > 0.45$). Mean observation time and range in this group was 195 days (range 12–489 days) respectively. The same calculation was made for the difference between tumour tissue and normal tissue and no significance was found between recurrence ($n = 26$) or disease-free survival ($n = 13$) ($P > 0.30$).

**Figure 2** EGFR immunoreactivity in head and neck squamous cell carcinomas ($n = 41$) as compared with results obtained for normal tissue from the same patient.

**Figure 3** The content of EGFR immunoreactivity in squamous cell carcinomas (mean 3.6; range 0.4–10.5 nmol $g^{-1}$ membrane protein), corresponding normal mucosa (mean 1.7; range 0.1–4.3 nmol $g^{-1}$ membrane protein), normal oral mucosa from patients with non-cancer diseases and normal mucosa from healthy individuals (mean 2.3; range 1.2–3.4 nmol $g^{-1}$ membrane protein) and salivary gland carcinomas (mean 2.3; range 0.7–4.1 nmol $g^{-1}$ membrane protein).

*Patients with malignant salivary gland carcinomas*

EGFRs measured in malignant salivary gland tumours demonstrated values similar to squamous cell carcinomas ($P > 0.1$) (Figure 3) (mean 2.3; range 0.7–4.1 nmol $g^{-1}$ membrane protein). These tumours were derived from the minor salivary glands and, as corresponding normal tissue was not obtainable, overexpression could not be investigated.

*Normal mucosa from patients and healthy individuals*

Analysis of receptor expression in normal tissue ($n = 41$) from patients with squamous cell carcinoma did not demonstrate a significantly higher level compared with healthy individual patients with non-cancer diseases ($n = 20$) (Figure 3). However, in the subgroup consisting of younger healthy individuals (mean age 29 years), EGFR expression was significantly higher (mean 2.5; range 1.2–3.4 nmol $g^{-1}$ membrane protein) compared with the patients' corresponding normal tissue (mean 1.7; range 0.1–4.3 nmol $g^{-1}$ membrane protein) ($P < 0.03$). In an age-matched group of individuals with non-cancer diseases ($n = 10$) the mean EGFR level was higher (mean 2.1; range 1.7–3.1 nmol $g^{-1}$ membrane protein) compared with the mean value obtained from the patients' normal mucosa (mean 1.7; range 0.1–4.3 nmol $g^{-1}$ membrane protein), but not significantly.

Patients' normal counterparts were also included in the analysis in order to evaluate if the EGFR level in surrounding non-diseased tissue reflected correlations with tumour size and/or clinical stage. However, no correlation could be determined. No significant difference in EGFR level was seen in normal epithelia between patients treated with primary surgery ($n = 13$) and patients receiving preoperative irradiation ($n = 28$) ($P > 0.25$).

### Discussion

It is well understood that growth regulation of normal cells is controlled in part by the interaction of growth factors produced by the cells or neighbouring cells and growth factor receptors present on the cells. Abnormal expression of growth factors and their receptors or abnormal responses to growth factors or both may be involved in cellular transformation and in the maintenance of the transformed phenotype (Cross and Dexter, 1991). EGFR is an important mitogenic molecule regarding epithelial cells, and overexpression seems to be a general motif for many malignant epithelial tumours. Therefore a number of studies quantitating the receptor in tumour specimens have been carried out in an attempt to define a molecule correlating with clinical parameters and/or clinical outcome, and such assays have indicated a clinical usefulness of quantitation of EGFRs in tumours such as gliomas, breast cancer and bladder cancer (Grimaux *et al.*, 1990; Neal *et al.*, 1990; Hurtt *et al.*, 1992). So far, little attention has been paid to the methodological aspects of receptor quantitation and results from different studies are difficult to compare. Most of the reported clinical studies have used radiolabelled ligand assays employing $^{125}$I-labelled EGF. Methodological variations such as processing of the tumour tissue to yield membrane preparation and multipoint or two-point binding assay could explain some discrepancies in the results, notably as regards the distribution of the levels (mean, median) and the indicated thresholds for 'positivity'. However, even if a standardised methodology could be employed the $^{125}$I-labelled EGF binding assay requires a relatively large amount of tissue (0.5–1 g) and is in general less precise than immunoassays.

We have developed an ELISA method which allows EGFR quantitation to be carried out on small tissue samples (20 mg). Our ELISA demonstrates overexpression of EGFR in 83% of head and neck tumour specimens. The mean values in our assay are high (mean 3.6; range 0.4–10.5 nmol $g^{-1}$ membrane protein) compared with two other studies using a similar assay for quantitation of EGFRs in breast carcinomas (mean 0.02; range 0.001–0.1 nmol $g^{-1}$ membrane protein and mean 0.006; range 0–0.2 nmol $g^{-1}$ membrane protein respectively) (Grimaux *et al.*, 1990; Spyratos *et al.*, 1994). One reason for the differences could be the method used for receptor extraction. We have used a method that prevents the association of EGFR with the actin filaments and a relatively long incubation time (12 h) with 2% triton X-100 (Hollenberg, 1990; van Bergen en Henegouwen *et al.*, 1992). Another reason may be the use of different calibration standards. So far no definitive/international/ WHO defined calibration standard is available. Finally it is of course possible that squamous cell carcinomas in general express more EGFRs than adenocarcinomas.

Besides these methodological and standardisation problems other aspects may be of importance. One of these is the stromal component, which is variable and which contributes to membrane protein and may be responsible for the wide range of receptor expression. This may explain why we found seven cases that expressed more EFGRs in the corresponding normal tissue than in the tumour (Figure 2) and may also serve as an explanation for the tumour specimen expressing an extremely high level of EGFR (76 nmol $g^{-1}$ membrane protein). Another factor in performing these studies is that the receptor may be expressed at different levels within the same tumour; in other words tumour heterogeneity at EGFR level. To overcome this problem the entire tumour has to be investigated, which is not possible in most cases.

Other studies have demonstrated overexpression in head and neck carcinomas (Ishitoya *et al.*, 1989; Kawamoto *et al.*, 1991; Santini *et al.*, 1991; Scambia *et al.*, 1991) (Table II). The methods used in these studies are Western blotting, radiolabelled ligand assay and dot blot (Table II). One important reason for the varying results may be the definition of normal tissue. Some of the studies used oral or laryngeal mucosa from healthy individuals (Scambia *et al.*, 1990; Kawamoto *et al.*, 1991). Another study used mucosa from both healthy individuals and placenta (Ishito *et al.*, 1989). The only study that used normal tissue from patients was that of Santini *et al.* (1991) and, in agreement with our study, they demonstrated overexpression in most tumours (Table II).

We have looked at prognostic value, tumour size and clinical stage as well as a number of other relevant parameters in an attempt to establish further clinicopathological correlations. However, within our sample set, no correlation was evident between overexpression and the examined parameters, although the mean values for EGFR increased with tumour size and advanced clinical stage.

In 1953 Slaughter *et al* published a classic report describing the novel concept they called 'field cancerisation'. This term referred to the basic pathogenic process that links the originally diagnosed tumours from head and neck cancer patients with other primary tumours in the oropharynx, larynx, oesophagus and lung. Our study comparing receptor quantities in normal mucosa from patients suffering from head and neck cancer with mucosa from healthy nonsmokers and patients with non-cancer diseases was performed in an attempt to investigate whether EGFR could serve as a marker for 'field cancerisation' or 'condemned mucosa syndrome' (Slaughter *et al.*, 1953). The results, however, did not demonstrate significantly more EGFRs in patients compared with the age-matched control group. Another study including oral mucosa from patients with head and neck carcinomas and from control patients without cancer has demonstrated, using Northern blot increased EGFR mRNA in the group of patients suffering from cancer (Grandis and Tweardy, 1992). These results may indicate that the receptor protein should be overexpressed as well. However, another study comparing EGF at mRNA and protein level in colon cancer cell lines did not demonstrate linearity between the transcription and translation product, indicating that not all mRNA may be translated to protein (Huang *et al.*, 1992). In summary this part of our study rejects the hypothesis of EGFR protein as a marker for 'field cancerisation' in normal mucosa and suggests that the overexpression first develops in later stages of carcinogenesis. Concerning oral dysplasia quantitative studies have yet to be performed, but immunohistochemical results have demonstrated expression of EGFR in all layers of the epithelium, results which may also indicate overexpression (Christensen *et al.*, 1992*a*).

The mechanism leading to increased expression of EGFR in head and neck carcinomas is not usually gene amplification, which is seen in only 5–20% of patients overexpressing the receptor and is not related to clinical outcome (Eisbruch *et al.*, 1987; Ishitoya *et al.*, 1989; Kearsley *et al.*, 1991; Leonard *et al.*, 1991; Furuta *et al.*, 1992; Irish and Bernstein, 1993). The major mechanism for overexpression thus develops post-transcriptionally and/or alternatively post-translationally. In a study including 17 specimens from head and neck carcinomas no amplification of mRNA was found, thus the mechanisms leading to overexpression are

**Table II** Studies quantitating EGF receptors in head and neck squamous cell carcinomas

| *Reference* | *Method* | *Normal tissue* | *Tumour tissue* | *Percentage of tumours with overexpression* |
|---|---|---|---|---|
| Ishitoya *et al.* (1989) | Western blot | Healthy individuals ($n = 2$) and placenta | $n = 21$ | 53 |
| Scambia *et al.* (1991) | Radiolabelled ligand assay | Healthy individuals ($n = 20$) | $n = 41$ | 50 |
| Kawamoto *et al.* (1991) | Dot blot | Healthy individuals ($n = 8$) | $n = 41$ | 50 |
| Santini *et al.* (1991) | Radiolabelled ligand assay | Corresponding normal tissue from patients ($n = 60$) | $n = 70$ | 98 |
| Present study | ELISA | Corresponding normal tissue from patients ($n = 41$) | $n = 41$ | 83 |
| | | Healthy age-matched individuals ($n = 10$) | $n = 54$ | 50 |

more likely mRNA stability and/or enhanced protease insensitivity (Eisbruch *et al.*, 1987). In accord with this hypothesis EGFR in A431, a cell line established from a vulva squamous cell carcinoma, appears to be degraded more slowly than in human fibroblasts (Wrann and Fox 1979, Krupp *et al.*, 1982), indicating enhanced protease insensitivity in malignant cells compared with normal cells.

Head and neck carcinomas have been investigated for other oncogene and proto-oncogene products besides EGFR (Merritt *et al.*, 1990; Kearsley *et al.*, 1991; Leonard *et al.*, 1991). The c-*erb*B-2 proto-oncogene product, which shares sequence homology with EGFR and which in breast cancer has been related to clinical outcome, has been found to be expressed in a very few to 50% of specimens from head and neck carcinomas and not related to clinical outcome (Schechter *et al.*, 1985; Kearsley *et al.*, 1991; Field *et al.*, 1992). One reason may be that this proto-oncogene product is expressed in particular in secretory cells and is therefore linked with adenocarcinomas (Gullick 1991).

The importance of EGFR determination for head and neck cancer remains a contentious issue and currently it is not possible to evaluate this fully, but overexpression of this mitogenic receptor seems to be a general motif for these types of tumour and may contribute to the unregulated or abberrant proliferation observed in the malignant phenotype. It has not been possible to establish significant clinicopathological correlations at EGFR level. One reason may be methodological aspects as mentioned above, another that the EGFR system consists of both the receptor and a group of different ligands, which also have to be elucidated, before a final statement concerning the clinical relevance of this system can be confirmed.

**Acknowledgements**

The authors are indebted to Mrs Inger-Marie Jensen for skilful technical assistance. The authors are also indebted to the Statistical Research Unit, University of Copenhagen for providing statistical assistance. The study was supported by the Danish Research Council, the Boel Foundation and the Danish Cancer Research Association.

### References

BARRANDON Y AND GREEN H. (1987). Cell migration is essential for sustained growth of keratinocyte colonies: the roles of transforming growth factor-α and epidermal growth factor. *Cell*, **50**, 1131–1137.

BAYER EA AND WILCHECK M. (1980). The use of avidin–biotin complex as a tool in molecular biology. *Methods Biochem. Anal.*, **26**, 1–45.

CHEN WS, LASAR CS, POENIE M, TSIEN RY, GILL GN AND ROSENFELD MG. (1987). Requirement for intrinsic protein tyrosine kinase in the immediate and late actions of the EGF receptor. *Nature*, **328**, 820–823.

CROSS M AND DEXTER TM. (1991). Growth factors in development, transformation, and tumorigenesis. *Cell*, **64**, 271–280.

CHRISTENSEN ME, THERKILDSEN MH, HANSEN BL, ALBECK H, HANSEN GN AND BRETLAU P. (1992*a*). Epidermal growth factor receptor expression on oral mucosa dysplastic epithelia and squamous cell carcinomas. *Eur. Arch. Otorhinolaryngol.*, **249**, 243–247.

CHRISTENSEN ME, THERKILDSEN MH, HANSEN BL, HANSEN GN AND BRETLAU P. (1992*b*). Immunohistochemical detection of epidermal growth factor receptor in laryngeal squamous cell carcinomas. *Acta Otolaryngol.*, **112**, 734–738.

DE LARCO JE AND TODARO GJ. (1978). Growth factors from murine sarcoma virus-transformed cells. *Proc. Natl Acad. Sci. U.S.A.*, **75**, 4001–4005.

DI MARCO E, PIERCE JH, FLEMING TP, KRAUS MH, MOLLOY CJ, AARONSON SA AND DI FIORE PP. (1989). Autocrine interaction between TGFα and the EGF-receptor: quantitative requirements for induction of the malignant phenotype. *Oncogene*, **4**, 831–838.

DOWNWARD J, YARDEN Y, MAYES E, SCRACE G, TOTTY N, STOCKWELL P, ULLRICH A, SCHLESSINGER J AND WATERFIELD MD. (1984). Close similarity of epidermal growth factor receptor and v-*erb*-B oncogene protein sequences. *Nature*, **307**, 521–527.

EISBRUCH A, BLICK M, LEE JS, SACKS PG AND GUTTERMAN J. (1987). Analysis of the epidermal growth factor receptor gene in fresh human head and neck tumors. *Cancer Res.*, **47**, 3603–3605.

ENGBAEK F. (1994). A general procedure for optimizing concentrations of capture antibody, biotinylated detecting antibody, and enzyme-labeled avidin in ELISAs: Application to assays for α-fetoprotein, prolactin, FSH and LH in serum. *J. Clin. Immunoassay*, **17**, 151–155.

FIELD JK, SPANDIDOS DA, YIAGNISIS M, GROSNEY JR, PAPADIMITRIOU K AND STELL PM. (1992). C-*erb*B-2 expression in squamous cell carcinoma of the head and neck. *Anticancer Res.*, **12**, 613–620.

FURUTA Y, TAKASU T, ASAI T, YOSHIMURA S, TOKUCHI F, SHINOHARA T, NAGASHIMA K AND INUYAMA Y. (1992). Clinical significance of the epidermal growth factor receptor gene in squamous cell carcinomas of the nasal cavities and paranasal sinuses. *Cancer*, **69**, 358–362.

GILL GN, KAWAMOTO T, COCHET C, LE A, SATO JD, MASUI H, McLEOD C AND MENDELSOHN J. (1984). Monoclonal anti-epidermal growth factor receptor antibodies which are inhibitors of epidermal growth factor binding and antagonists of epidermal growth factor-stimulated tyrosine protein kinase activity. *J. Biol. Chem.*, **259**, 7755–7760.

GRANDIS JR AND TWEARDY DJ. (1992). The role of peptide growth factors in head and neck carcinoma. *Otolaryngol. Clin. N. Am.*, **25**, 1105–1115.

GRIMAUX M, MADY E, REMVIKOS Y, LAINE-BIDRON C AND MAGDELE NAT H. (1990). A simplified immuno-enzymetric assay of the epidermal growth factor receptor in breast tumours: Evaluation in 282 cases. *Intl. J. Cancer*, **45**, 255–262.

GULLICK WJ. (1991). Prevalence of aberrant expression of the epidermal growth factor receptor in human cancers. *Br. Med. Bull.*, **47**, 87–98.

GUSTERSON B, COWLEY G, SMITH JA AND OZANNE B. (1984). Cellular localisation of human epidermal growth factor receptor. *Cell Biol. Int. Rep.*, **8**, 649–658.

HIGASHIYAMA S, ABRAHAM JA, MILLER J, FIDDES JC AND KLAGSBRUN M. (1991). A heparin-binding growth factor secreted by macrophage-like cells that is related to EGF. *Science*, **251**, 936–939.

HOCK RA, NEXØ E AND HOLLENBERG MD. (1980). Solubilization and isolation of the human placenta receptor for epidermal growth factor-urogastrone. *J. Biol. Chem.*, **255**, 10737–10743.

HOLLENBERG MD. (1990). Receptor solubilization, characterization, and isolation. In *Methods in Neurotransmitter Receptor Analysis*, Yamamura, HI (ed.) pp. 111–145. Raven Press: New York.

HUANG S, TRUJILLO JM AND CHAKRABARTY S. (1992). Proliferation of human colon cancer cells: Role of epidermal growth factor and transforming growth factorα. *Intl. J. Cancer*, **52**, 978–986.

HURTT MR, MOOSSY J, DONOVAN-PELUSO M AND LOCKER J. (1992). Amplification of epidermal growth factor receptor gene in gliomas: Histopathology and prognosis. *J. Neuropathol. Exp. Neurol.*, **51**, 84–90.

IRISH JC AND BERNSTEIN A. (1993). Oncogenes in head and neck cancer. *Laryngoscope*, **103**, 42–52.

ISHITOYA J, TORIYAMA M, OGUCHI N, KITAMURA K, OHSHIMA M, ASANO K AND YAMAMOTO T. (1989). Gene amplification and overexpression of EGF receptor in squamous cell carcinomas of the head and neck. *Br. J. Cancer*, **59**, 559–562.

JØRGENSEN PE, POULSEN SS AND NEXØ E. (1988). Distribution of i.v. administered epidermal growth factor in the rat. *Reg. Peptides*, **23**, 161–169.

KAWAMOTO T, TAKAHASHI K, NISHI M, KIMURA T, MATSUMURA T AND TANIGUCHI S. (1991). Quantitative assay of epidermal growth factor receptor in human squamous cell carcinomas of the oral region by an avidin–biotin method. *Jpn. J. Cancer Res.*, **82**, 403–410.

KEARSLEY JH, LENOARD JH, WALSH MD AND WRIGHT GR. (1991). A comparison of epidermal growth factor receptor (EGFR) and c-*erb*B-2 oncogene expression in head and neck squamous cell carcinomas. *Pathology*, **23**, 189–194.

KISSANE JM. (1990). *Andersons Pathology*, vol. 1, pp. 602, CV Mosby: St Louis.

KRAUS MH, ISSING W, MIKI T, POPESCU NC AND AARONSON SA. (1989). Isolation and characterization of *ERBB3*, a third member of the *ERBB*/epidermal growth factor receptor family: Evidence for overexpression in a subset of human mammary tumors. *Proc. Natl Acad. Sci. USA*, 86, 9193–9197.

KRUPP MN, CONNOLLY DT AND LANE MD. (1982). Synthesis, turnover and down regulation of epidermal growth factor receptors in human A431 epidermoid carcinoma cells and skin fibroblasts. *J. Biol. Chem.*, **257**, 11489-11496.

LEONARD JH, KEARSLEY JH, CHENEVIX-TRENCH G AND HAYWARD NK. (1991). Analysis of gene amplification in head-and-neck squamous-cell carcinomas. *Intl J. Cancer*, **48**, 511-515.

MANGILI R, KEMENY DM, LI LK AND VIBERTY GC. (1987). Development of a sensitive enzyme-linked immunosorbent assay (ELISA) for quantitation of human IgG subclasses. *J. All. Clin. Immun.*, **79**, 223.

MASSAGUE J AND PANDIELLA A. (1993). Membrane-anchored growth factors. *Annu. Rev. Biochem.*, **62**, 515-541.

MERRIT WD, WEISSLER MC, TURK BF AND GILMER TM. (1990). Oncogene amplification in squamous cell carcinoma of the head and neck. *Arch. Otolaryngol. Head Neck Surg.*, **116**, 1394-1398.

NANNEY LB, MCKANNA JA, STOSCHECK CM, CARPENTER G & KING LE. (1984). Visualization of epidermal growth factor receptors in human epidermis. *J. Invest. Dermatol.*, **82**, 165-169.

NEAL DE, SHARPLES L, SMITH K, FENNELLY J, HALL RR, HARRIS AL. (1990). The epidermal growth factor receptor and the prognosis of bladder cancer. *Cancer*, **65**, 1619-1625.

NEXØ E AND HANSEN HF. (1985). Binding of epidermal growth factor from man, rat and mouse to the human epidermal growth factor receptor. Biochem. Biophys. Acta, **843**, 101-106.

NEXØ E, HOCK RA AND HOLLENBERG MD. (1979). Lectin-agarose immobilization, a new method for detecting soluble membrane receptors. *J. Biol. Chem.*, **254**, 8740-8743.

NICHOLSON S, SAINSBURY JRC, NEEDHAM GK, CHAMBERS P, FARNDON JR AND HARRIS AL. (1988). Quantitative assays of epidermal growth factor receptor in human breast cancer: Cut-off points of clinical relevance. *Intl. J. Cancer*, **42**, 36-41.

OZANNE B, SHUM A, RICHARDS CS, CASSELLS D, GROSSMAN D, TRENT J, GUSTERSON B AND HENDLER F. (1986). Evidence for an increase of EGF receptors in epidermoid malignancies. *Cancer Cells*, **3**, 41-49.

OZAWA S, UEDA M, ANDO N, SHIMIZU N AND ABE O. (1989). Prognostic significance of epidermal growth factor receptor in esophageal squamous cell carcinomas. *Cancer*, **63**, 2169-2173.

PLOWMAN GD, CULOUSCOU JM, WHITNEY GS, GREEN JM, CARLTON GW, FOY L, NEUBAUER MG AND SHOYAB M. (1993). Ligand-specific activation of HER4/p180$^{erbB4}$, a fourth member of the epidermal growth factor receptor family. *Proc. Natl Acad. Sci.*, 90, 1746-1760.

REINSCH CH. (1967). Smoothing by spline functions. *Numer. Math.*, **10**, 177-183.

RIEDEL H, MASSOGLIA S, SCHLESSINGER J AND ULLBRICH A. (1988). Ligand activation of overexpressed epidermal growth factor receptors transforms NIH 3T3 mouse fibroblasts. *Proc. Natl Acad. Sci. USA*, **85**, 1477-1481.

SANTINI J, FORMENTO J, FRANCOUAL M, MILANO G, SCHNEIDER M, DASSONVILLE O AND DEMARD F. (1991). Characterization, quantification and potential clinical value of the epidermal growth factor receptor in head and neck squamous cell carcinomas. *Head and Neck*, **13**, 132-139.

SATO JD, KAWAMOTO T, LE AD, MENDELSOHN J, POLIKOFF J AND SATO GH. (1983). Biological effects *in vitro* of monoclonal antibodies to human epidermal growth factor receptors. *Mol. Biol. Med.*, **1**, 511-529.

SASADA R, ONO Y, TANIYAMA Y, SHING Y, FOLKMAN J AND IGARASHI K. (1993). Cloning and expression of cDNA encoding human betacellulin, a new member of the EGF family. *Biochem. Biophys. Res. Commun.*, **190**, 1173-1179.

SCAMBIA G, PANICI PB, BATTAGLIA F, FERRANDINA G, ALMADORI G, PALUDETTI G, MAURIZI M AND MANCUSO S. (1991). Receptors for epidermal growth factor and steroid hormones in primary laryngeal tumors. *Cancer*, **67**, 1347-1351.

SCHECHTER AL, HUNG M, VAIDYANATHAN L, WEINBERG RA, YANGFENG TL, FRANCKE U, ULLRICH A. & COUSSENS L. (1985). The *neu* gene: An *erb*B-homologous gene distinct from and unlinked to the gene encoding the EGF receptor. *Science*, **229**, 976-978.

SHOYAB M, PLOWMAN GD, MCDONALD VL, BRADLEY JG AND TODARO GJ. (1989). Structure and function of human amphiregulin: A member of the epidermal growth factor family. *Science*, **243**, 1074-1076.

SLAUGHTER DP, SOUTHWICK HW AND SMEJKAL W. (1953). 'Field cancerization' in oral stratified squamous epithelium: Clinical implications of multicentric origin. *Cancer*, **6**, 963-968.

SPIESSL B, BEAHRS OH, HERMANEK P, HUTTER RVP, SCHEIBE O, SOBIN LH AND WAGNER G. (1990). *TNM Atlas Illustrated Guide to the TNM/TNM Classification of Malignant Tumours*, 3rd edn. Springer: Berlin.

SPYRATOS F, MARTIN PM, HACENE K, ANDRIEU C, ROMAIN S, FLOIRAS JL AND MAGDELENAT H. (1994). Prognostic value of a solubilized fraction of EGF receptors in a primary breast cancer using an immunoenzymatic assay a retrospective study. *Breast Cancer Res. Treat.*, **29**, 85-95.

STAMPFER MR, PAN CH, HOSODA J, BARTHOLOMEW J, MENDELSOHN J AND YASWEN P. (1993). Blockage of EGF receptor signal transduction causes reversible arrest of normal and immortal human mammary epithelial cells with synchronous reentry into the cell cycle. *Exp. Cell Res.*, **208**, 175-188.

STROOBANT P, RICE AP, GULLICK WJ, CHENG DJ, KERR IM AND WATERFIELD MD. (1985). Purification and characterization of vaccinia virus growth factor. *Cell*, **42**, 383-393.

VAN BERGEN EN HENEGOUWEN PMP, DEN HARTIGH JC, ROMEYN P, VERKLEIJ AJ AND BOONSTRA J. (1992). The epidermal growth factor receptor is associated with actin filaments. *Exp. Cell Res.*, **199**, 90-97.

VAN DAM PA, LOWE DG, WATSON JV, JAMES M, CHARD T, HUDSON CN AND SHEPHERD JH. (1991). Multiparameter flow-cytometric quantitation of epidermal growth factor receptor and c-erbB-2 oncoprotein in normal and neoplastic tissues of the female genital tract. *Gyneocol. Oncol.*, **42**, 256-264.

WATERFIELD MD, MAYES ELV, STROOBANT P, BENNET PLP, YOUNG S, GOODFELLOW PN, BANTING GS AND OZANNE B. (1982). A monoclonal antibody to the epidermal growth factor receptor. *J. Cell Biochem.*, **20**, 149-161.

WRANN MM AND FOX CF. (1979). Identification of epidermal growth factor receptors in a hyperproducing human epidermoid carcinoma cell line. *J. Biol. Chem.*, **254**, 8083-8086.

YASUI W, SUMIYOSHI H, HATA J, KAMEDA T, OCHIAI A, ITO H AND TAHARA E. (1988). Expression of epidermal growth factor receptor in human gastric and colonic carcinomas. *Cancer Res.*, **48**, 137-141.